# High failure rate in *Pseudomonas aeruginosa*-associated periprosthetic hip and knee joint infections

**DOI:** 10.5194/jbji-11-83-2026

**Published:** 2026-02-10

**Authors:** Ece Akcicek, Jennyfer A. Mitterer, Veronika Achatz, Tamino Szirmay, Sujeesh Sebastian, Jochen G. Hofstaetter

**Affiliations:** 1 Michael Ogon Laboratory for Orthopaedic Research, Orthopaedic Hospital Speising, Vienna, Austria; 2 Center for Anatomy and Cell Biology, Medical University of Vienna, Vienna, Austria; 3 Department of Orthopaedics and Trauma, Medical University of Graz, Graz, Austria; 4 2nd Department, Orthopaedic Hospital Speising, Vienna, Austria

## Abstract

**Introduction**: *Pseudomonas aeruginosa* is one of the most common Gram-negative (GN) pathogens in periprosthetic joint infections (PJIs). However, not many data are available on them. This study aimed to evaluate the prevalence of *P. aeruginosa* in hip and knee PJIs, patient characteristics, types of infection, resistance patterns, treatments, and outcomes. **Methods**: We retrospectively analyzed culture-positive revision total hip and knee arthroplasties (rTHA and rTKA) from 2008 to 2023. Cases were evaluated according to the International Consensus Meeting (ICM) 2018 and European Bone and Joint Infection Society (EBJIS) criteria 2021. The success rate was calculated according to Tier classification. **Results**: Among 1640 revision procedures performed on 1286 patients, 195 revisions in 160 patients were positive for GN microorganisms, including 50 *P. aeruginosa* cases (3.1 %, hip/knee: 39/11) in 38 patients. Most were chronic (64 %), monomicrobial (74 %) infections, particularly infected rTHA (63.8 %). *Proteus mirabilis* was the main co-pathogen (23.1 %) in polymicrobial infections. The mean follow-up time was 65.4 months. The most frequent surgical intervention was two-stage exchange (48 %). Chronic infections required significantly more total revisions than acute cases. Success rates decreased with each additional revision. Antibiotic resistance developed in three patients during subsequent revisions. The overall success rate was 46 %. Reinfection-free survival decreased from 95 % at 12 months to 22.4 % after 10 years. **Conclusion**: In our patient cohort, *Pseudomonas aeruginosa* accounted for one-third of hip and knee GN PJIs, was mostly found in infected rTHA, and was monomicrobial. Changes in antimicrobial resistance, high failure rates, and low long-term infection-free survival underline that *Pseudomonas aeruginosa* is a challenging PJI pathogen.

## 1 Introduction

Periprosthetic joint infections (PJIs) represent a severe complication following total joint arthroplasty (TJA). Gram-negative (GN) PJIs are on the rise, with the literature reporting incidences of up to 23 % (Hsieh et al., 2009; Sujeesh et al., 2018; Tattevin et al., 1999; Zimmerli et al., 2004). PJIs caused by GN organisms are considered more challenging due to their growing resistance to antibiotics, increased risk of subsequent re-revisions, and their presence in highly comorbid patients, which is linked to worse outcomes (Hsieh et al., 2009; Zmistowski et al., 2011).

*Pseudomonas aeruginosa* is an aerobic, biofilm-producing GN bacteria. It is identified as one of the most prevalent GN pathogens associated with hip and knee PJIs, accounting for approximately 20 %–40 % of cases (Hsieh et al., 2009; Lyczak et al., 2000; Rodríguez-Pardo et al., 2014; Zmistowski et al., 2011). Several studies have investigated *P. aeruginosa*-associated PJIs. Cerioli et al. (2020) emphasized the difficulty in treating implant-associated infections, which require prolonged intravenous and oral antibiotics. Kim et al. (2024) reported variable infection clearance rates after DAIR and two-stage revisions. Prié et al. (2022) observed favorable outcomes (82 %) in chronic, monomicrobial ciprofloxacin-susceptible PJIs treated with one-stage exchange, whereas Shah et al. (2016) found worse outcomes in patients managed with DAIR. Liu et al. (2025) noted that *P. aeruginosa* is not more difficult to treat than other GN bacteria in cases of acute infections when it is treated with DAIR. Although previous studies provide valuable insights into *P. aeruginosa*-associated PJIs, the results remain heterogeneous, limited by non-standardized outcome reporting tools and definitions with variable follow-up duration. Moreover, there is a paucity of data on whether antibiotic resistance can evolve in patients undergoing one or more subsequent re-revisions with *P. aeruginosa*-positive cultures.

The primary aim of this study was to analyze the prevalence of *P. aeruginosa* in hip and knee PJIs over a 16-year period. The secondary aims were to determine clinical characteristics, surgical interventions, the microbial spectrum of co-pathogens, antibiotic treatment, and changes in the antibiotic resistance patterns in patients undergoing subsequent *P. aeruginosa*-positive re-revisions and to assess the re-revision free survival.

## 2 Methods and materials

### 2.1 Study design and population

After the institution's research ethics board approval (EK 10/2020), a retrospective single-center analysis was conducted from our prospectively maintained arthroplasty and PJI databases. We analyzed a total of 1640 culture-positive hip and knee revision arthroplasties (rTHA/rTKA) performed on 1286 patients between January 2008 and December 2023. All patients who underwent septic or aseptic rTHA/rTKA with a positive intraoperative culture for *P. aeruginosa* were included for the final analysis.

Cases were classified based on the International Consensus Meeting (ICM) 2018 (Shohat et al., 2019) and the European Bone and Joint Infection Society (EBJIS) criteria (Mcnally et al., 2021). Patient-specific risk factors were evaluated using the McPherson classification (Coughlan and Taylor, 2020) and Charlson Comorbidity Index (CCI) (Roffman et al., 2016). PJIs were classified as acute or chronic based on onset of symptoms after primary or revision arthroplasty: acute if within 3 months and chronic if after 3 months (Zimmerli et al., 2004). Presumed aseptic revisions that yielded positive cultures from intraoperative samples were classified as unexpected positive intraoperative cultures (UPICs). Furthermore, infected cases were defined as infected primary TJA if the infection occurred after primary implantation or as infected revision TJA if the infection occurred after aseptic revision surgery.

### 2.2 Follow-up and clinical outcome

The mean follow-up time was 65.4±38 months, after excluding the follow-up duration of the five patients who died within that period from the calculation. All subsequent re-revisions with a positive intraoperative culture of *P. aeruginosa* were included. Tier classification was used to access the clinical outcome at a minimum follow-up of 1 year and ranged from Tier 1 to 4. Infection control without the need for continued antibiotic therapy or subsequent re-revisions was classified as “Tier 1”. Continuous suppressive antibiotic treatment was designated as “Tier 2”, while any requirement for surgical intervention or spacer retention was defined as “Tier 3”. Mortality occurring within less than 1 year (4A) or more than 1 year (4B) from the initiation of PJI treatment was classified as “Tier 4” (Abblitt et al., 2019). Tier 1 and Tier 4B were accepted as successful as long as the death was unrelated to PJI.

### 2.3 Microbiological analysis

Routine microbiological analysis was conducted on periprosthetic tissue samples, intraoperative swabs, and implant sonication fluid during all revision surgeries. The median number of cultures obtained per surgery was 4.5 (range: 1–10) with a median number of two (range: 1–9) positive *P. aeruginosa* results. Swabs were considered positive cultures only when obtained intraoperatively. Explanted devices were immediately placed into sonication containers, to which saline solution was added to cover the implants completely. The container was then vortexed and sonicated. Periprosthetic tissue samples, intraoperative swabs, and sonication fluids (0.1 mL) were analyzed for bacteria and fungi using standard microbiological techniques, as described previously (Frank et al., 2021). Antimicrobial susceptibility profiling was determined using the BD system (Becton Dickinson and Company, Franklin Lakes, NJ) according to the manufacturer's recommendations and interpreted following the European Committee on Antimicrobial Susceptibility Testing guidelines.

### 2.4 Statistical analysis

Continuous variables were assessed for normality using Shapiro–Wilk tests and presented as means ( M ) ± standard deviations (SD) or medians (MD) with interquartile ranges (IQR), displayed as the first ( $Q_{1}$ ) and third ( $Q_{3}$ ) quartiles. Comparisons were performed using the Mann–Whitney U test. Categorical variables were analyzed using the $\chi^{2}$ test of independence and Fisher's exact test. A binary logistic regression model was performed to identify independent predictors of treatment success. For all tests, a p value of <0.05 was considered significant. The Kaplan–Meier method was used to estimate reinfection-free survival rate with 95 % confidence intervals (CIs). Statistical analyses were performed with IBM, SPSS version 26.0 statistic software (SPSS Inc., Chicago, IL).

## 3 Results

Overall, 195/1640 (11.9 %; hip/knee: 139/56) procedures of 160/1286 (12.4 %) patients had a positive intraoperative culture for a GN pathogen. Of these, 50 revision arthroplasties (25.6 %, hip/knee: 39/11) performed on 38 patients (23.8 %, female/male: 27/11) yielded *P. aeruginosa* in intraoperative cultures and were included in the final analysis.

From 50 *P. aeruginosa*-positive procedures, 47 (94 %) of them were associated with PJI. Infections were acute in 13/47 cases (27.7 %) and chronic in 34/47 (72.3 %). Among these infections, 12/47 (25.5 %) procedures were infected primary TJAs and 35/47 (74.5 %) were infected revision TJAs. A total of 37 cases (74 %) were identified as monomicrobial, with the remaining 13 cases (26 %) being polymicrobial. *P. aeruginosa* was identified in three presumed aseptic revisions (6 %, hip/knee: 2/1). Detailed patient demographics, infection types, and classifications are presented in Table 1.

**Table 1:** Patient demographics and classifications according to the International Consensus Meeting 2018, European Bone and Joint Infection Society 2021, Charlson CI (Charlson Comorbidity Index), and McPherson score. Bold values indicate clinically relevant subgroup classifications. These include positive intraoperative cultures, categorized as either UPIC or infected cases, and the Charlson Comorbidity Index, which highlights patient comorbidities.

	Culture-positive revision TJA		
	(2008–2023)		
	Proc.: 1640		
	Hip/knee: 971/669		
	Pat.: 1286		
	*Pseudomonas aeruginosa*		
	Positive revision TJA		
	Proc. (%): 50/1640 (3.1)		
	Pat. (%): 38/1286 (3)		
	Total	Hip	Knee
	Proc: 50	Proc: 39/50 (78 %)	Proc: 11/50 (22 %)
	Pat: 38	Pat: 28/38 (73.7 %)	Pat: 10/38 (26.3 %)
Male/female	11/27	7/21	4/6
Age in yrs. (avg ± SD)	70±12	69±12.8	71±9.7
BMI (avg ± SD)	31.9±6.1	32.7±5.9	29.6±6
UPIC (%)	**3** (**6**)	**2** (**4**)	**1** (**2**)
Septic (%)	**47** (**94**)	**37** (**74**)	**10** (**20**)
Acute PJI (%)	13 (27.7)	9 (19.2)	4 (8.5)
Chronic PJI (%)	34 (72.3)	28 (59.6)	6 (12.8)
Inf. Primary TJA (%)	12 (25.5)	7 (14.9)	5 (10.6)
Inf. Revision TJA (%)	35 (74.5)	30 (63.8)	5 (10.6)
Monomicrobial (%)	37 (74)	29 (58)	8 (16.0)
Polymicrobial (%)	13 (26)	10 (20)	3 (6.0)
ICM 2018			
Infected (%)	40 (80)	34 (68)	6 (12)
Inconclusive (%)	8 (16)	4 (8)	4 (8)
Not infected (%)	2 (4)	1 (2)	1 (2)
EBJIS 2021			
Confirmed (%)	36 (72)	29 (58)	7 (14)
Likely (%)	14 (28)	10 (20)	4 (8)
Unlikely (%)	0 (0)	0 (0)	0 (0)
Charlson CI, mdn ± SD	4.1±2.3	4.4±2.5	3.7±2.1
McPherson score			
– Infection grade			
I	13	9	4
II	3	2	1
III	34	28	6
– Systemic host grade			
A	13	10	3
B	29	22	7
C	8	7	1
– Local extremity grade			
1	10	6	4
2	27	20	7
3	13	13	0
Tier classification			
1 (%)	15 (30)	12 (24)	3 (6)
2 (%)	0 (0)	0 (0)	0 (0)
3 (%)	18 (36)	14 (28)	4 (8)
4 (%)	17 (28)	13 (26)	4 (8)

TJA: total joint arthroplasty, SD: standard deviation, UPIC: unexpected positive intraoperative culture, PJI: prosthetic joint infection, Proc: procedure, Pat: patient, n : number, BMI: body mass index, avg: average, mdn: median.

### 3.1 Microbiological analysis and co-infections

A total of 225 samples were obtained from 50 revision procedures, of which 177 (78.7 %) yielded a positive intraoperative culture. Among 144/177 positive cultures, *P. aeruginosa* was identified. Distributions of the microbiological spectrum are displayed in Table 2.

**Table 2:** Detailed total microbiological sample types and counts. The data are categorized by hip and knee, including samples with co-infections.

	Overall	Hip				Knee		
		Infected	Infected	UPIC		Infected	Infected	UPIC
		primary	revision			primary	revision	
		TJA	TJA			TJA	TJA	
Microbiologies total (%)	225 (100)	45 (20)	133 (59.1)	4 (1.8)		19 (8.4)	17 (7.6)	7 (3.1)
Tissue sample total (%)	114 (50.6)	19 (8.4)	72 (32)	0 (0)		11 (4.9)	6 (2.7)	6 (2.7)
Swab intraoperative (%)	87 (38.7)	21 (9.3)	49 (21.8)	4 (1.8)		5 (2.2)	8 (3.6)	0 (0)
Sonication total (%)	24 (10.7)	5 (2.2)	12 (5.3)	0 (0)		3 (1.3)	3 (1.3)	1 (0.4)
Total positive samples (%)	177 (78.7)	44 (19.6)	100 (44.4)	4 (1.8)		15 (6.7)	9 (4)	5 (2.2)
Pos. cultures for *P. aeruginosa* (%)	144 (64)	44 (19.6)	79 (35.1)	4 (1.8)		8 (3.6)	8 (3.6)	1 (0.4)
Tissue sample (%)	59 (26.2)	19 (8.4)	35 (15.6)	0 (0)		3 (1.3)	1 (0.4)	1 (0.4)
Swab intraoperative (%)	69 (30.7)	20 (8.9)	37 (16.4)	4 (1.8)		3 (1.3)	5 (2.2)	0 (0)
Sonication (%)	16 (7.1)	5 (2.2)	7 (3.1)	0 (0)		2 (0.9)	2 (0.9)	0 (0)
Detected microorganism (%)	200 (100)	44 (22)	111 (55.5)	4 (2)		23 (11.5)	9 (4.5)	9 (4.5
In monomicrobial (%)	119 (59.5)	44 (22)	57 (28.5)	4 (2)		7 (3.5)	7 (3.5)	(0)
In polymicrobial (%)	81 (35.5)	0 (0)	54 (27)	0 (0)		16 (8)	2 (1)	9 (4.5)
*P. aeruginosa* in polymicrobial infections (%)	25 (12.5)	0 (0)	22 (11)			1 (0.5)	1 (0.5)	1 (0.5)
Detected co-pathogens (%)	56 (100)	0 (0)	32 (57.1)			15 (26.8)	1 (1.8)	8 (14.3)
*GN Pathogens*								
*Pseudomonas species*	1 (1.8)		1 (1.8)					
*Proteus mirabilis*	6 (10.7)		6 (10.7)					
*Escherichia coli*	2 (3.6)		2 (3.6)					
*Citrobacter koseri*	1 (1.8)		1 (1.8)					
*Bacteroides fragilis*	4 (7.1)							4 (7.1)
*Enterobacter cloacae*	8 (14.3)					8 (4)		
*GP Pathogens*								
*Staphylococcus haemolyticus*	2 (3.6)		2 (3.6)					
*Beta-hemolytic Streptococci (Gr. A)*	6 (10.7)		6 (10.7)					
*Enterococcus faecalis*	6 (10.7)		6 (10.7)					
*Staphylococcus epidermidis*	2 (3.6)		2 (3.6)					
*Bacillus cereus*	1 (1.8)							1 (1.8)
*Finegoldia magna*	4 (7.1)					4 (7.1)		
*Propionibacterium propionicum*	1 (1.8)					1 (1.8)		
*Cutibacterium avidum*	2 (3.6)		3 (1.5)			2 (3.6)		
*Staphylococcus aureus*	3 (1.5)							3 (1.5)
*Fungi*								
*Candida parapsilosis*	1 (1.8)						1 (1.8)	
*Candida dubliniensis*	3 (1.5)		3 (1.5)					

PJI: periprosthetic joint infection, TJA: total joint arthroplasty, GN: Gram-negative, GP: Gram-positive, UPIC: unexpected intraoperative cultures, Gr.: group.

Among the 13 polymicrobial cases, intraoperative cultures identified two different pathogens in 9 cases (69.2 %), four pathogens in 1 case (7.7 %), and five pathogens in 3 cases (23.1 %). *Proteus mirabilis* was the most frequently observed pathogen in co-infections, found in 3 cases (23.1 %), all of which were rTHA. The distribution of concomitant microorganisms is shown in Table 3.

**Table 3:** Microorganisms that identified alongside *Pseudomonas aeruginosa* in polymicrobial infections.

	Combination with first	Combination with second	Combination with third	Combination with fourth
	microorganism	microorganism	microorganism	microorganism
*rTHA*	*Proteus mirabilis*	–	–	–
*rTHA*	*Candida dubliniensis*	–	–	–
*rTHA*	*Staphylococcus haemolyticus*	–	–	–
*rTHA*	*Beta-hemolytic Streptococcus (Group A)*	–	–	–
*rTHA*	*Enterococcus faecalis*	–	–	–
*rTHA*	*Cutibacterium avidum*	–	–	–
*rTHA*	*Proteus mirabilis*	–	–	–
*rTKA*	*Candida parapsilosis*	–	–	–
*rTHA*	*Pseudomonas species*	–	–	–
*rTHA*	*Staphylococcus haemolyticus*	–	–	–
*rTHA*	*Proteus mirabilis*	–	–	–
*rTHA*	*Bacteroides fragilis*	–	–	–
*rTHA*	*Finegoldia magna*	*Escherichia coli*	*Staphylococcus epidermidis*	*Enterococcus faecalis*
*rTHA*	*Candida parapsilosis*	*Escherichia coli*	*Staphylococcus epidermidis*	*Citrobacter koseri*
*rTKA*	*Pseudomonas species*	*Staphylococcus aureus*	*Bacillus cereus*	–
*rTKA*	*Staphylococcus haemolyticus*	*Enterobacter cloacae*	*Cutibacterium avidum*	*Propionibacterium propionicum*

Poly- or monomicrobial infections had no significant impact on outcomes ( p=0.509 ). Polymicrobial cases were mostly chronic (9/13) and identified during rTHA (10/13) but showed no significant association with either rTHA ( p=0.554 ) or chronicity ( p=0.930 ).

### 3.2 Resistance pattern and treatment

**Figure 1:**
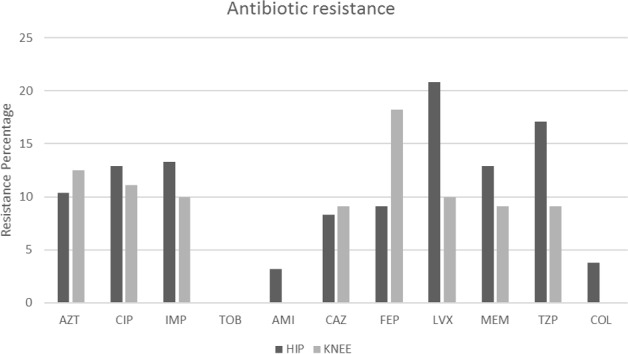
Antibiotic resistance percentages in *Pseudomonas aeruginosa* antibiograms in revision total hip and knee arthroplasties (AZT = aztreonam, CIP = ciprofloxacin, IMP = imipenem, TOB = tobramycin, AMI = amikacin, CAZ = ceftazidime, FEP = cefepime, LVX = levofloxacin, MEM = meropenem, TZP = piperacillin/tazobactam, COL = colistin).

No resistance to tobramycin was observed during antimicrobial susceptibility testing in all cases of culture-positive *P. aeruginosa*. The highest antibiotic resistance was observed among quinolones, with levofloxacin showing a resistance rate of 17.7 %, followed by piperacillin/tazobactam (15.2 %). The resistance rates of the tested antibiotics and their distribution within rTHA and rTKA are presented in Fig. 1. Multidrug-resistant Gram-negative bacteria (MDR-GNB) were detected in 5/50 cases (10 %).

One patient experienced multiple recurrent episodes of PJI, including two episodes caused by *P. aeruginosa*, with a period of culture negativity between the episodes. Therefore, a total of 39 treatment procedures are described for 38 patients.

The most frequently employed treatment was a combination of ciprofloxacin and meropenem (13/39), with an average duration of 54.8 days (mean: 60.4 d, range: 15 to 140 d). This was followed by meropenem monotherapy (6/39), which had an average duration of 61.1 d. The remaining patients received heterogenous treatment combinations including piperacillin/tazobactam, imipenem, moxifloxacin, and gentamicin.

Two patients developed complications during their antibiotic therapy. The first patient, who was receiving meropenem and ciprofloxacin, developed leukopenia on postoperative day 46, leading to the discontinuation of ciprofloxacin. The second patient developed leukopenia on postoperative day 22 while receiving treatment with ciprofloxacin, piperacillin/tazobactam, and meropenem. All antibiotics were subsequently discontinued. Detailed durations and treatment modalities are available in the supplementary table.

### 3.3 Changes in the antibiotic resistance pattern during subsequent re-revisions

Multiple revisions with positive *P. aeruginosa* culture were identified in 8 (21.1 %) patients. All susceptibility changes were observed in cases of rTHA. The first patient received ceftolozane/tazobactam monotherapy, during which resistance to cefepime developed; concurrently, the patient was receiving caspofungin for the treatment of *Candida dubliniensis* infection. Meropenem monotherapy was administered to the second patient, who subsequently developed resistance to aztreonam. The third patient was likewise treated with meropenem monotherapy; resistance to ceftazidime, cefepime, meropenem, and piperacillin/tazobactam developed. The fourth patient received dual therapy with ciprofloxacin, imipenem, and piperacillin/tazobactam. Resistance initially developed to imipenem, followed by additional resistance to aztreonam, ciprofloxacin, meropenem, and piperacillin/tazobactam during continued ciprofloxacin and piperacillin/tazobactam therapy.

Polymicrobial infections were found in 3/8 patients. Table 4 provides detailed information on these patients, including the surgical site, the type of revision procedure performed, the type of infection, the time between revisions, and the changes observed in the resistance profile.

**Table 4:** Overview of patients with positive intraoperative *Pseudomonas aeruginosa* cultures during multiple revisions. Every line represents one patient. Surgical site, type of revision procedure, type of infection, antibiotic treatment, mean time between revisions, and changes in resistance profiles are presented. Notably, antibiotics to which the isolate has developed resistance are written in bold.

Surgical site	Revision 1 and resistances	Mono/poly	Treatment	Subsequent re-revision 1 and resistances	Mono/poly	Treatment	Subsequent re-revision 2 and resistances	Mono/poly	Treatment	Subsequent re-revision 3 and resistances	Mono/poly	Treatment
rTHA	Single-stage	Mono	CIP + MEM	Two-stage	Mono	CIP + MEM	–	–	–	–	–	–
*rTHA*	DAIR	Mono	TZP + MFX	Two-stage	Mono	MEM	DAIR R: CAZ, MEM, TZP, FEP	Mono	GEN + CIP	–	–	–
*rTHA*	DAIR R: CAZ, MEM, TZP, IMP.	Poly	C/T + CAS	DAIR R: CAZ, MEM, TZP, IMP, **FEP**.	Mono	C/T + CAS	–	–	**–**	–	–	**–**
*rTKA*	DAIR	Mono	MFX + MEM	DAIR	Mono	MFX + MEM	–	–	–	–	–	–
*rTHA*	Spacer exchange	Mono	MFX + CXM	DAIR R: CIP, LVX	Mono	MEM	Two-stage R: **AZT**, CIP, LVX	Mono	MEM	DAIR R: AZT, CIP, LVX.	Mono	MEM
*rTHA*	Two-stage	Poly	CIP + IMP + TZP	DAIR R: **IMP**	Mono	CIP + TZP	Two-stage R: **AZT**, **CIP**, **MEM**, **TZP**, IMP	Mono	CIP^*^	–	–	–
*rTHA*	DAIR	Mono	CIP + IMP	DAIR	Mono	CIP + IMP	–	–	–	–	–	
*rTHA*	DAIR	Poly	MEM + MFX	DAIR	Poly	MEM	–	**–**	–	—	**–**	
Mean time between revisions in days (SD)	–			40.3 (50.5)			23 (14.7)			19 (0)		

^*^Although the patient was resistant to ciprofloxacin, treatment with the drug proceeded due to the delayed arrival of the test results, which were received only after treatment had begun. Unfortunately, the patient died within 1 week (rTHA = revision total hip arthroplasty, rTKA = revision total knee arthroplasty, Pat = patient, mono = monomicrobial infection, poly = polymicrobial infection, DAIR = debridement, antibiotics, and implant retention; R = resistant, AZT = aztreonam, CIP = ciprofloxacin, IMP = imipenem, CAZ = ceftazidime, FEP = cefepime, LVX = levofloxacin, MEM = meropenem, TZP = piperacillin/tazobactam, MFX = moxifloxacin, CXM = cefuroxime, C/T = ceftolozane/tazobactam, CAS = caspofungin, GEN = gentamicin).

### 3.4 Surgical procedures and outcome 

**Figure 2:**
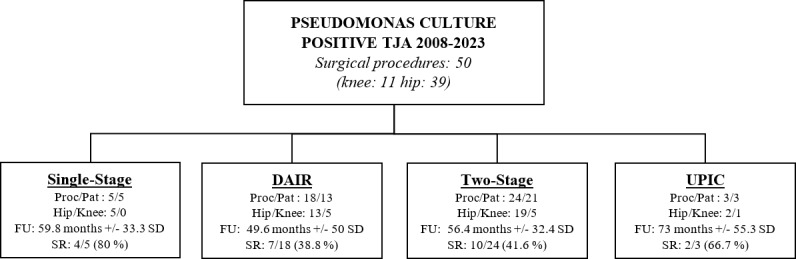
Surgical procedures that patients underwent for *Pseudomonas* PJI. Success rate calculated according to the MSIS Outcome Reporting Tool (MSIS ORT). Tier 1 and 4B accepted as successful. Proc/Pat = procedure count/patient count, TJA: total arthroplasty, FU: follow-up duration, SR: success rate, DAIR: debridement, antibiotics, and implant retention, UPIC: unexpected intraoperative cultures.

The revision surgeries comprised 18/50 (36 %) debridement, antibiotics, and implant retention (DAIR) procedures; 24 (48 %) two-stage revisions; five single-stage revisions (10 %); and three UPIC procedures (6 %). UPICs were identified in two aseptic stem/cup exchange procedures and in one single-stage revision performed due to mechanical complications. Two-stage revisions include resection arthroplasty and spacer implantation. Surgical procedures are detailed in Fig. 2.

Overall, the success rate was 46 % (23/50), considering only Tier 1 and 4B cases to be successful (15/50 Tier 1 and 8/50 Tier 4B). No cases were classified as Tier 2, while 18 were classified as Tier 3 and 9 as Tier 4A; all of these were considered failures. Of the 27 failed procedures (54 %), 4 were after rTKA and 23 were after rTHA. In septic cases, the highest success rate was achieved in single-stage procedures (4/5, 80 %), whereas two-stage procedures (10/24, 41.6 %) had the poorest outcome. Of the 18 cases treated with DAIR, 13 had chronic infections, with a success rate of 38.4 % (5/13), while 5 of them had acute infections with a success rate of 60 % (3/5). In the UPIC group, there was 1 failed case due to a septic complication, subsequently treated with resection arthroplasty.

A decrease in success rates was observed as the number of revision surgeries increased ( p<0.001 ). The median number of overall required revision surgeries was 5 (range: 1–13). A spacer retention or salvage-type procedure (resection arthroplasty: 13, amputation: 6) was required for infection control during follow-up in 19 of the 50 procedures. The number of chronic infections and total revisions was significantly higher in the re-revision group compared to the primary revision ( p<0.001 ). The infected revision group underwent an average of four subsequent revisions. Similarly, chronic infections were associated with a greater total number of revisions ( p<0.001 ), with an average of three additional procedures.

**Figure 3:**
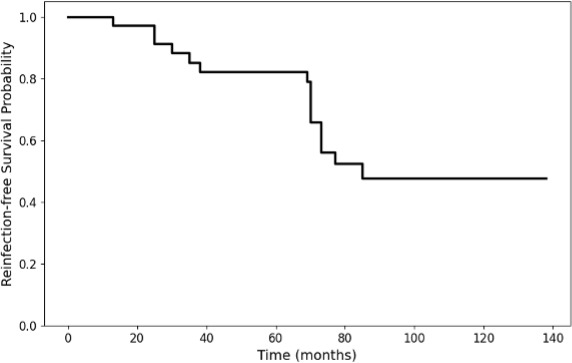
Kaplan–Meier survival curve shows the reinfection-free survival probability after *Pseudomonas aeruginosa*-associated infection in revision total hip and knee arthroplasty.

Figure 3 illustrates the reinfection-free survival probability over time. Median time was 85 months. The rate was 100 %, 85 %, 82 %, and 48 % following a 1-, 3-, 5-, and 10-year follow-up period.

## 4 Discussion

We analyzed data from 38 patients who underwent a total of 50 hip and knee revision arthroplasties with a positive intraoperative culture for *P. aeruginosa*. Overall, *P. aeruginosa*-associated PJIs presented with considerable complexity, showed changing antibiotic resistance patterns in patients with subsequent re-revisions, and were characterized by high rates of treatment failure. Remarkably, the majority of the cases were rTHA (78 %) and a significant proportion of these cases were infected revision TJAs, accounting for 70 %. Moreover, *P. aeruginosa*-associated PJIs were predominantly chronic and monomicrobial.

PJI cases caused by GN infection, including *P. aeruginosa*, have been mostly documented as acute and polymicrobial (Cerioli et al., 2020; Hsieh et al., 2009). However, our study mainly identified chronic and monomicrobial infections, similar to the findings of Prié et al. (2022). This observation may be related to the high proportion of revision TJAs within our cohort and aligns with the known strong biofilm-forming capacity of *P. aeruginosa*, which has been reported to hinder the detection of co-pathogens. Moreover, our study predominantly used conventional diagnostic methods, which, as previous studies have suggested, may preferentially detect fast-growing, dominant microorganisms. This may potentially mask slower-growing, anaerobic microorganisms that are difficult to culture, resulting in an underestimation of true polymicrobial diversity (Dhawan et al., 2016; Patnaik et al., 2025; Donlan, 2005; Trampuz et al., 2007).

Prior surgical interventions represent another key factor contributing to infection persistence (Frank et al., 2021; Mitterer et al., 2022; Siegert et al., 2023). Such prior procedures often compromise soft tissue quality, weaken the local immune response, and thereby increase susceptibility to PJI, particularly in revision TJAs (Zimmerli et al., 2004). Consistently, previous aseptic revisions have been associated with higher PJI incidence compared to primary TJAs (Lenguerrand et al., 2017). In line with this, our study showed that infected revision cases had a significantly higher incidence of chronic infection and greater total number of required revisions compared to revisions of primary TJAs. Our findings underline the persistent and recurrent nature of *P. aeruginosa* in PJIs, with multiple subsequent revisions with positive cultures.

Polymicrobial infections were most commonly observed in chronic cases and rTHA surgeries. Previous studies have also shown that polymicrobial infections and GN bacteria are more prevalent in rTHA than in rTKA (Stevoska et al., 2022; Tsai et al., 2019). These findings may be explained by differences in surgical exposure and soft tissue coverage. Furthermore, *P. mirabilis* was the most commonly observed co-pathogen in polymicrobial infections. This differs remarkably from other studies in which the dominant concomitant organisms were *Staphylococcus aureus* (Shah et al., 2016), GP cocci (Prié et al., 2022), and unspecified GP organisms (Kim et al., 2024). However, a recent study reported frequent concomitance of *P. aeruginosa* and *Proteus mirabilis* (Achatz et al., 2025). The high rate of rTHA in our study, along with the anatomical proximity of the urinary system to the hip joint and their shared vascular and lymphatic networks, may explain the frequent occurrence of concomitant *P. mirabilis* infections.

The most common treatment regimen was a combination of meropenem and ciprofloxacin. This therapeutic approach is consistent with the literature, which advocates for the use of a prolonged β -lactam antibiotic, such as meropenem, in combination with ciprofloxacin for susceptible strains (Le Vavasseur and Zeller, 2022). Notably, fluoroquinolones are known to have the high rate of biofilm permeability and bactericidal activity, particularly against *P. aeruginosa* (Abdi-Ali et al., 2006). Among fluoroquinolones, ciprofloxacin is the only oral antibiotic with reliable activity against *P. aeruginosa* and the ability to penetrate the deeper layers of the biofilms (Anderl et al., 2000; Walters et al., 2003). This is supported by a recent study, which found that a minimum of 3 months of ciprofloxacin monotherapy was associated with improved outcomes (Cerioli et al., 2020). However, other studies did not observe any significant superiority among various antibiotic regimens (Shah et al., 2016; Tekes-Manuva et al., 2024). In our study, significance testing was not performed due to heterogeneous antibiotic regimens, precluding direct comparison with these findings. Nevertheless, the high failure rate observed in our cohort suggests that achieving reliable treatment success in *P. aeruginosa* PJI remains a persistent challenge despite existing recommendations.

Two-stage procedure is recommended for patients with chronic PJIs, compromised soft tissue conditions, and/or infections caused by DTR (difficult-to-treat) microorganisms (Zimmerli et al., 2004). In our study, the majority of procedures were performed as two-stage revisions, reflecting the high prevalence of chronic infections. Although *P. aeruginosa* is generally considered to be a DTR pathogen, a recent study by Liu et al. (2025) suggests that early acute PJI caused by *P. aeruginosa* does not present greater treatment challenges than other GN PJIs when treated with DAIR. However, previous studies have shown high DAIR failure rates for chronic GN infections (Duque et al., 2017; Zhu et al., 2021). According to the latest EBJIS recommendations (Sigmund et al., 2025), DAIR should not be performed in cases of chronic infection. However, due to the retrospective design of this study, the included DAIR procedures were performed on a case-by-case basis by the surgeon, taking into account the patient's condition and the surgeon's preference before standardized in-house protocols were established.

An adequate follow-up period is essential for accurately capturing changes in resistance patterns and outcomes, as short follow-ups may fail to detect late microbiological shifts, low-grade infections with failed infection control, or persistent infections with undetected pathogens. To our knowledge, this is the first study reporting changes in antibiotic resistance patterns in patients with more than one culture-positive *P. aeruginosa* re-revision with a long follow-up. Previous studies have described microbial profile and resistance changes during up to one following re-revision or two-stage exchange procedure in a non-microorganism-specific manner and focusing on other implant-related infections (Akgün et al., 2017; Frank et al., 2021; Kim et al., 2024; Kurd et al., 2010; Mitterer et al., 2022; Tan et al., 2016). Since changes in microbial resistance may lead to treatment failure (Frank et al., 2021), comprehending the microorganism-specific resistance dynamics in chronic hip and knee infections with long-term follow-up is essential.

Given the retrospective design of our study, several limitations must be acknowledged. This study includes data before a standardized in-house protocol for intraoperative sample harvesting was established, resulting in a relatively high number of swabs used for microbiological analysis. This may have led to variations and inaccuracies in pathogen identification. However, we only accepted swabs taken intraoperatively as a diagnostic tool until 2018. Additionally, defining success based on surgical outcomes does not reflect patients' clinical complaints, quality of life, and overall health status. Furthermore, as our study is single-centered, it may be influenced by specific patient demographics, comorbidities, and local antibiotic resistance patterns. Due to the small cohorts in subgroup analyses, the statistical power may be limited; therefore results should be interpreted with caution.

## 5 Conclusion

In conclusion, *P. aeruginosa*-related PJIs are common among GN PJIs and clinically challenging due to their high failure rate. Our findings show that PJIs associated with *P. aeruginosa* occur primarily in infected revision hip arthroplasties and are usually chronic and monomicrobial. Infection duration and total revision count significantly affect the clinical outcomes. Changes in antibiotic resistance patterns in patients with subsequent *P. aeruginosa*-positive re-revisions highlight the necessity of comprehensive diagnostics and reflect the limitation of treatment options in achieving successful infection control in *P. aeruginosa*-associated PJIs.

## Supplement

10.5194/jbji-11-83-2026-supplement The supplement related to this article is available online at https://doi.org/10.5194/jbji-11-83-2026-supplement.

## Data Availability

The data of this study are available upon reasonable request.

## References

[bib1.bib1] Abblitt WP, Ascione T, Bini S, Bori G, Brekke AC, Chen AF, Courtney PM, Della Valle CJ, Diaz-Ledezma C, Ebied A, Fillingham YJ, Gehrke T, Goswami K, Grammatopoulos G, Marei S, Oliashirazi A, Parvizi J, Polkowski G, Saeed K, Schwartz AJ, Segreti J, Shohat N, Springer BD, Suleiman LI, Swiderek LK, Tan TL, Yan CH, Zeng YR (2019). Hip and Knee Section, Outcomes: Proceedings of International Consensus on Orthopedic Infections. J Arthroplasty.

[bib1.bib2] Abdi-Ali A, Mohammadi-Mehr M, Agha Alaei Y (2006). Bactericidal activity of various antibiotics against biofilm-producing Pseudomonas aeruginosa. Int J Antimicrob Agents.

[bib1.bib3] Achatz V, Mitterer JA, Huber S, Akcicek E, Tobudic S, Sebastian S, Hofstaetter JG (2025). Proteus-species-associated periprosthetic hip and knee joint infections – a 15-year cohort analysis. J Bone Joint Infect.

[bib1.bib4] Akgün D, Müller M, Perka C, Winkler T (2017). Positive bacterial culture during re-implantation is associated with a poor outcome in two-stage exchange arthroplasty for deep infection. Bone Joint J.

[bib1.bib5] Anderl JN, Franklin MJ, Stewart PS (2000). Role of Antibiotic Penetration Limitation in Klebsiella pneumoniae Biofilm Resistance to Ampicillin and Ciprofloxacin. Antimicrob Agents Chemother.

[bib1.bib6] Cerioli M, Batailler C, Conrad A, Roux S, Perpoint T, Becker A, Triffault-Fillit C, Lustig S, Fessy MH, Laurent F, Valour F, Chidiac C, Ferry T (2020). Pseudomonas aeruginosa Implant-Associated Bone and Joint Infections: Experience in a Regional Reference Center in France. Front Med (Lausanne).

[bib1.bib7] Coughlan A, Taylor F (2020). Classifications in Brief: The McPherson Classification of Periprosthetic Infection. Clin Orthop Relat Res.

[bib1.bib8] Dhawan B, Sebastian S, Malhotra R, Kapil A, Gautam D (2016). Prosthetic joint infection due to Lysobacter thermophilus diagnosed by 16S rRNA gene sequencing. Indian J Med Microbiol.

[bib1.bib9] Donlan RM (2005). New approaches for the characterization of prosthetic joint biofilms. Clin Orthop Relat Res.

[bib1.bib10] Duque AF, Post ZD, Lutz RW, Orozco FR, Pulido SH, Ong AC (2017). Is There Still a Role for Irrigation and Debridement With Liner Exchange in Acute Periprosthetic Total Knee Infection?. J Arthroplasty.

[bib1.bib11] Frank BJH, Aichmair A, Simon S, Schwarz GM, Dominkus M, Hofstaetter JG (2021). Analysis of Culture Positive First and Second Stage Procedures in Periprosthetic Knee and Hip Joint Infections. J Arthroplasty.

[bib1.bib12] Hsieh PH, Lee MS, Hsu KY, Chang YH, Shin HN, Ueng SW (2009). Gram-negative prosthetic joint infections: risk factors and outcome of treatment. Clin Infect Dis.

[bib1.bib13] Kim BI, Schwartz AM, Wixted CM, Prado IP, Polascik BA, Seidelman JL, Seyler TM (2024). Outcomes After Pseudomonas Prosthetic Joint Infections. J Am Acad Orthop Surg.

[bib1.bib14] Kurd MF, Ghanem E, Steinbrecher J, Parvizi J (2010). Two-stage exchange knee arthroplasty: Does resistance of the infecting organism influence the outcome?. Clin Orthop Relat Res.

[bib1.bib15] Lenguerrand E, Whitehouse MR, Beswick AD, Jones SA, Porter ML, Blom AW (2017). Revision for prosthetic joint infection following hip arthroplasty: Evidence from the National Joint Registry. Bone Joint Res.

[bib1.bib16] Le Vavasseur B, Zeller V (2022). Antibiotic Therapy for Prosthetic Joint Infections: An Overview. Antibiotics.

[bib1.bib17] Liu W-Y, Hendriks JGE, van Kempen RWTM, van der Weegen W, Rijnen WHC, Goosen JHM, van der Zwaard BC, Pronk Y, Zijlstra WP, ten Have BLEF, Ploegmakers JJW, Wouthuyzen-Bakker M (2025). Clinical Outcome of Patients with Acute Periprosthetic Joint Infections Caused by Pseudomonas aeruginosa Compared to Other Gram-Negative Bacilli. Microorganisms.

[bib1.bib18] Lyczak JB, Cannon CL, Pier GB (2000). Establishment of Pseudomonas aeruginosa infection: lessons from a versatile opportunist. Microbes Infect.

[bib1.bib19] Mcnally M, Sousa R, Wouthuyzen-Bakker M, Chen AF, Soriano A, Vogely HC, Clauss M, Higuera CA, Trebše R (2021). The EBJIS definition of periprosthetic joint infection. Bone Joint J.

[bib1.bib20] Mitterer JA, Frank BJH, Gardete-Hartmann S, Panzenboeck LF, Simon S, Krepler P, Hofstaetter JG (2022). Changes of the microbiological spectrum and antibiotic resistance pattern in postoperative spinal implant infections with multiple culture-positive revision surgeries. Spine J.

[bib1.bib21] Patnaik A, Kayal T, Basu S (2025). Polymicrobial Infections: A Comprehensive Review on Current Context, Diagnostic Bottlenecks and Future Directions. Acta Microbiol Hellen.

[bib1.bib22] Prié H, Meyssonnier V, Kerroumi Y, Heym B, Lidove O, Marmor S, Zeller V (2022). Pseudomonas aeruginosa prosthetic joint-infection outcomes: Prospective, observational study on 43 patients. Front Med (Lausanne).

[bib1.bib23] Rodríguez-Pardo D, Pigrau C, Lora-Tamayo J, Soriano A, del Toro MD, Cobo J, Palomino J, Euba G, Riera M, Sánchez-Somolinos M, Benito N, Fernández-Sampedro M, Sorli L, Guio L, Iribarren JA, Baraia-Etxaburu JM, Ramos A, Bahamonde A, Flores-Sánchez X, Corona PS, Ariza J, Amat C, Larrosa MN, Puig M, Murillo O, Cabo X, Goenaga MÁ, Elola M, De la Herrán G, Garcia-Arenzana JM, García-Ramiro S, Martínez-Pastor JC, Tornero E, García-Lechuz JM, Marín M, Villanueva M, López I, Cisterna R, Santamaría JM, Gómez MJ, Puente A, Cano P, Horcajada JP, González-Mínguez P, Portillo E, Puig L, Franco M, Jordán M, Coll P, Amador-Mellado J, Fuster-Foz C, García-Paíno L, Nieto I, Muniain MÁ, Suárez AI, Praena J, Gómez MJ, Puente A, Maseguer MA, Garagorri E, Pintado V, Marinescu C, Ramírez A, Montaner F, Múñez E, Álvarez T, García R, Puente E, Salas C, Fariñas MC, Pérez JM, Achabal BV, Montejo Baranda JM (2014). Gram-negative prosthetic joint infection: outcome of a debridement, antibiotics and implant retention approach. A large multicentre study. Clin Microbiol Infect.

[bib1.bib24] Roffman CE, Buchanan J, Allison GT (2016). Charlson Comorbidities Index. J Physiother.

[bib1.bib25] Shah NB, Osmon DR, Steckelberg JM, Sierra RJ, Walker RC, Tande AJ, Berbari EF (2016). *Pseudomonas* Prosthetic Joint Infections: A Review of 102 Episodes. J Bone Joint Infect.

[bib1.bib26] Shohat N, Bauer T, Buttaro M, Budhiparama N, Cashman J, Della Valle CJ, Drago L, Gehrke T, Marcelino Gomes LS, Goswami K, Hailer NP, Han SB, Higuera CA, Inaba Y, Jenny JY, Kjaersgaard-Andersen P, Lee M, Llinás A, Malizos K, Mont MA, Jones RM, Parvizi J, Peel T, Rivero-Boschert S, Segreti J, Soriano A, Sousa R, Spangehl M, Tan TL, Tikhilov R, Tuncay I, Winkler H, Witso E, Wouthuyzen-Bakker M, Young S, Zhang X, Zhou Y, Zimmerli W (2019). Hip and Knee Section, What is the Definition of a Periprosthetic Joint Infection (PJI) of the Knee and the Hip? Can the Same Criteria be Used for Both Joints?: Proceedings of International Consensus on Orthopedic Infections. J Arthroplasty.

[bib1.bib27] Siegert P, Frank BJH, Simon S, Meraner D, Pokorny-Olsen A, Diepold J, Wurnig C, Hofstaetter JG (2023). Changes in microbiological spectrum and antibiotic susceptibility in two-stage exchange for periprosthetic shoulder infections. Arch Orthop Trauma Surg.

[bib1.bib28] Sigmund IK, Ferry T, Sousa R, Soriano A, Metsemakers W-J, Clauss M, Trebse R, Wouthuyzen-Bakker M (2025). Debridement, antimicrobial therapy, and implant retention (DAIR) as curative strategy for acute periprosthetic hip and knee infections: a position paper of the European Bone & Joint Infection Society (EBJIS). J Bone Joint Infect.

[bib1.bib29] Stevoska S, Himmelbauer F, Stiftinger J, Stadler C, Pisecky L, Gotterbarm T, Klasan A (2022). Significant Difference in Antimicrobial Resistance of Bacteria in Septic Revision between Total Knee Arthroplasty and Total Hip Arthroplasty. Antibiotics.

[bib1.bib30] Sujeesh S, Malhotra R, Sreenivas V, Kapil A, Chaudhry R, Dhawan B (2018). Sonication of orthopaedic implants: A valuable technique for diagnosis of prosthetic joint infections – ScienceDirect. J Microbiol Meth.

[bib1.bib31] Tan TL, Gomez MM, Manrique J, Parvizi J, Chen AF (2016). Positive culture during reimplantation increases the risk of subsequent failure in two-stage exchange arthroplasty. J Bone Joint Surg.

[bib1.bib32] Tattevin P, Crémieux A-C, Pottier P, Huten D, Carbon C (1999). Prosthetic Joint Infection: When Can Prosthesis Salvage Be Considered?. Clin Infect Dis.

[bib1.bib33] Tekes-Manuva D, Babich T, Kozlovski D, Elbaz M, Yahav D, Halperin E, Leibovici L, Avni T (2024). What is the most effective antibiotic monotherapy for severe Pseudomonas aeruginosa infection? A systematic review and meta-analysis of randomized controlled trials. Clin Microbiol Infect.

[bib1.bib34] Trampuz A, Piper KE, Jacobson MJ, Hanssen AD, Unni KK, Osmon DR, Mandrekar JN, Cockerill FR, Steckelberg JM, Greenleaf JF, Patel R (2007). Sonication of Removed Hip and Knee Prostheses for Diagnosis of Infection. N Engl J Med.

[bib1.bib35] Tsai Y, Chang CH, Lin YC, Lee SH, Hsieh PH, Chang Y (2019). Different microbiological profiles between hip and knee prosthetic joint infections. J Orthop Surg.

[bib1.bib36] Walters MC, Roe F, Bugnicourt A, Franklin MJ, Stewart PS (2003). Contributions of antibiotic penetration, oxygen limitation, and low metabolic activity to tolerance of Pseudomonas aeruginosa biofilms to ciprofloxacin and tobramycin. Antimicrob Agents Chemother.

[bib1.bib37] Zhu MF, Kim K, Cavadino A, Coleman B, Munro JT, Young SW (2021). Success Rates of Debridement, Antibiotics, and Implant Retention in 230 Infected Total Knee Arthroplasties: Implications for Classification of Periprosthetic Joint Infection. J Arthroplasty.

[bib1.bib38] Zimmerli W, Trampuz A, Ochsner PE (2004). Prosthetic-Joint Infections. N Engl J Med.

[bib1.bib39] Zmistowski B, Fedorka CJ, Sheehan E, Deirmengian G, Austin MS, Parvizi J (2011). Prosthetic Joint Infection Caused by Gram-Negative Organisms. J Arthroplasty.

