# Supplementary material for: High failure rate in Pseudomonas aeruginosa-associated periprosthetic hip and knee joint infections
Source: J Bone Jt Infect. 2026 Feb 10;11(1):83–94. doi: 10.5194/jbji-11-83-2026 (PMC12917492; doi:10.5194/jbji-11-83-2026)
Supplement: The supplement related to this article is available online at https://doi.org/10.5194/jbji-11-83-2026-supplement. [file jbji-11-83-2026-supplement.pdf]

Supplement of J. Bone Joint Infect., 11, 83–94, 2026
https://doi.org/10.5194/jbji-11-83-2026-supplement
© Author(s) 2026. CC BY 4.0 License.

JBJI
Journal of Bone and Joint Infection
Open Access

Supplement of

High failure rate in *Pseudomonas aeruginosa*-associated periprosthetic hip and knee joint infections

Ece Akcicek et al.

Correspondence to: Jochen G. Hofstaetter (researchlab@oss.at)

The copyright of individual parts of the supplement might differ from the article licence.

Pat.	Joint	Concomitant organism	Antibiotic treatment	Duration (Weeks)	Tier class.
1	rTHA		Meropenem	6	4B
2	rTKA		Meropenem + Ciprofloxacin	6	1
3	rTHA	*Proteus mirabilis*	Meropenem + Ciprofloxacin	6	1
4	rTHA		Meropenem + Ciprofloxacin (2 weeks) -> Meropenem (4 weeks)	6	3D
5	rTHA		Piperacillin/Tazobactam + Moxifloxacin (3 weeks) -> Meropenem (4 weeks) -> Gentamycin + Ciprofloxacin (8 weeks)	15	3D
6	rTHA		Ceftriaxon+ Moxifloxacin (2 weeks) -> Meropenem + Ciprofloxacin (6 weeks)	8	1
7	rTHA	*Candida dubliniensis*	Ceftolozan/Tazobactam and Caspofungin	8	4A
8	rTKA		Moxifloxacin (2 weeks) + Meropenem (12 weeks)	12	4B
9	rTHA		Meropenem + Ciprofloxacin	10	3D
10	rTHA		Ciprofloxacin + Piperacillin/Tazobactam	22 days*	4B
11	rTHA		Meropenem	20	4A
12	rTHA	*Staphylococcus homolyticus*	Linezolid (4 weeks) + Meropenem (20 days)	4	1
13	rTKA		Ceftriaxon + Moxifloxacin (2 weeks) -> Gentamycin (2 weeks) + Ciprofloxacin (15 weeks)	17	3D
14	rTKA		Ciprofloxacin (2 weeks) + Piperacillin/Tazobactam (5 weeks)	7	4B
15	rTHA	*Beta Hemolytic Streptococcus (Group A)*	Ceftriaxon + Ciprofloxacin	4	4B
16	rTKA		Meropenem + Ciprofloxacin	4	3D
17	rTHA		Meropenem + Ciprofloxacin	4	4B
18	rTHA		Ciprofloxacin + Piperacillin/Tazobactam (7 weeks) -> Meropenem + Ciprofloxacin (6 weeks)	13	1
19	rTHA		Meropenem	3	1
20	rTHA	*Enterococcus faecalis*	Moxifloxacin + Ceftriaxon (2 weeks) -> Gentamycin + Ampicillin + Meropenem + Ciprofloxacin (3 weeks) -> Ciprofloxacin (9 weeks)	14	1
21	rTHA	*Cutibacterium avidum*	Ceftriaxon + Meropenem + Moxifloxacin (2 weeks) -> Piperacillin/Tazobactam + Moxifloxacin (2 weeks) -> Ciprofloxacin (5 weeks)	9	3D
22	rTHA		Moxifloxacin (4 weeks) + Cefuroxim (2 weeks) -> Meropenem (8 weeks) -> Tobramycin + Ceftolozan/Tazobactam (4 weeks)	16	3D
23	rTHA		Meropenem + Ciprofloxacin	3	3D
24	rTHA		Piperacillin/Tazobactam + Ciprofloxacin (5 days)-> Ciprofloxacin (4 weeks)	5	1
25	rTHA		Piperacillin/Tazobactam	3	4A
26	rTHA	*Proteus mirabilis*	Meropenem + Ciprofloxacin (2 weeks) -> Ciprofloxacin (2 weeks)	4	1
27	rTHA		Meropenem + Ciprofloxacin (6 weeks) -> Ciprofloxacin (6 weeks)	12	1
28	rTKA	*Candida parapsilosis*	Meropenem + Flucanozol (2 weeks) -> Ciprofloxacin + Flucanozol (6 weeks)	8	3E

29	rTHA		Meropenem + Ciprofloxacin (6 weeks) -> Meropenem (12 weeks)	18**	1
30	rTHA	*Pseudomonas species*	Ciprofloxacin (16 weeks) + Imipenem (10 weeks) + Piperacillin/Tazobactam (11 weeks)	16	4A
31	rTHA		Imipenem + Ciprofloxacin	5	4A
32	rTKA		Meropenem	12	3E
33	rTHA		Meropenem	9	3D
34	rTHA		Ciprofloxacin + Piperacillin/Tazobactam (5 days) -> Ciprofloxacin (6 weeks)	7	1
35	rTHA	*Enterococcos faecalis, Staphylococcus homolyticus, Staphylococcus epidermidis, Escherichia coli*	Meropenem (3 weeks) + Doxycyclin (3 weeks) Moxifloxacin (1 week) -> Ciprofloxacin + Amoxicillin-Clavulanate (9 weeks)	12	3E
36	rTHA	*Proteus mirabilis,* Citrobacter *koseri Staphylococcus epidermidis, Escherichia coli*	Meropenem + Teicoplanin	2	3E
37	rTKA	*Bacteroides fragilis, Staphylococcus aereus, Bacillius cereus*	Cefuroxim + Moxifloxacin (1 week) -> Ciprofloxacin (4 weeks)	5	1
38	rTKA	*Finegoldia magna. Enterobacter clocae, Cutibacterium avidium, Propionibacterim propionicum*	Cefuroxim + Meropenem (3 weeks) -> Ciprofloxacin (4 weeks)	7	1
39	rTKA		Meropenem+Ciprofloxacin	14	4B

Table S1: Detailed antibiotic therapy, duration, surgical side and Tier classification of the patients with a positive *Pseudomonas aeruginosa* culture. Each line represents one patient. Patients 35 and 36 refer to the same patient but they are listed separately because patient had two episodes of PJI, with a period of culture negativity in between.

*Antibiotic therapy was discontinued on postoperative day 22 due to the development of leukopenia.

** The patient developed leukopenia on postoperative day 46.

(rTHA = revision total hip arthroplasty, rTKA = revision total knee arthroplasty)
